# Spatio-temporal evolution of the resilience of Chinese border cities

**DOI:** 10.3389/fpubh.2022.1101799

**Published:** 2022-12-20

**Authors:** Lili Sui, Fei Peng, Siyu Wu

**Affiliations:** ^1^School of Geography, Liaoning Normal University, Dalian, China; ^2^Center for Korean Peninsula Studies, Liaodong University, Dandong, China; ^3^Center for Studies of Marine Economy and Sustainable Development, Liaoning Normal University, Dalian, China; ^4^Institute of Marine Sustainable Development, Liaoning Normal University, Dalian, China

**Keywords:** border cities, spatio-temporal pattern, urban resilience, regional differences, Chinese prefecture-level cities, China border region

## Abstract

In China, border cities are developing in the direction of trade, investment, tourism, and regional diversification and becoming crucial for the national opening-up strategy and inter-regional exchange. In this study, we construct a comprehensive system for measuring and evaluating the resilience of border cities in China that also reveals the spatial and temporal characteristics of resilience. Three representative sample zones (Northeast, Northwest, and Southwest) are selected within the three major regions of China to analyze the regional differences in border city resilience and propose targeted coping strategies. The findings of this study are as follows. First, the spatial distribution of resilience in Chinese border cities varies significantly, with the overall resilience decreasing in the following order: Northeast China > Southwest China > Northwest China > North China > Tibetan China. Higher resilience of border cities is predominantly related to better economic foundations and advantages in border trade. Second, the resilience of China's border cities has increased significantly over the past decade, with highly resilient border cities concentrated in the northeastern part of China, the northern part of Xinjiang, and Guangxi Province. Moreover, high resilience generally spreads to surrounding low-resilience cities over time. Third, the spatial distribution and development trends of resilience levels differ among the three sample zones. Therefore, it is crucial to improve urban resilience according to the regional characteristics of each border city and its specific developmental stage.

## 1. Introduction

With further globalization of the Chinese economy, urbanization, industrialization, and informatization are increasingly spreading to the country's border areas. Thus, frontiers that served as barriers in earlier times no longer exist. Cities that have evolved from settlements based on their unique locations, typically considered border cities, now take on the dual functions of national security barriers and economic and cultural links (1). Since the introduction of the Belt and Road Initiative, China's border cities have strengthened their roles in economic exchange, trade activities, and population movements in the process of opening the country to the outside world (2). In this context, the State Council and the Ministry of Commerce have issued a series of policies to support economic development and urban construction in border areas. However, because of their natural resources and location, border cities are more fragmented and weaker than mainland and coastal cities in terms of the transport of goods, information transfer, and cultural exchange. Border cities that develop in a frontier environment are exposed to a greater variety of uncertainties and unknown risks, as well as more significant potential negative impacts and consequences (3, 4). Therefore, optimizing the development of China's border cities involves two main aspects: (1) increasing the resilience of border cities in response to unpredictable shocks and (2) mitigating the negative impacts of external and internal disruptions to the greatest possible extent.

Research on border cities has long been a key issue in political geography (5–7). Most studies focus on cross-border trade and investment in developed countries (8), the “twin cities” model of symbiotic development (9), community management in small border cities (10), ethnicity and refugees (11), terrorism, and drug trafficking under non-traditional security (12); therefore, relatively little attention has been paid to developing countries. Within China, research has focused on economic development, trade activities, population movements, urbanization levels, ethnic culture, and tourism development at the border (13–17). Advances in border city research have also been mutually absorbing and complementary, despite vast differences in terms of research perspectives, scales, focus, and methodologies. Notably, regarding non-traditional security and global cooperation across regions and borders, countries have paid more attention to the development of border cities, as well as the origins of urbanization and its special development process in typical regions, including border cities (18, 19). Currently, qualitative analysis such as policy interpretation and historical evolution is often used to analyze the commonalities of Chinese border cities or the specificities of a particular city (20, 21). Conversely, quantitative studies construct evaluation index systems for border cities to measure their development status and driving forces, which compensates for the lack of research on the development issues of domestic border cities from the perspective of border location conditions (16, 22). Owing to historical development differences and geographical constraints, the development of China's border cities shows clear spatial imbalances. Coupled with crises in recent years, such as terrorist activities in neighboring countries, refugee problems caused by war, and the risk of importing infectious diseases, the issue of border city governance has become increasingly urgent. Therefore, applying the concept of resilience to the study of Chinese border cities has profound theoretical and practical value.

Collectively, little research has been conducted on the development quality and status of China's border cities, with most studies on urban resilience focusing on national or regional scales, such as cities in the central-eastern and coastal regions of China; thus, border cities have long been neglected (23). Moreover, the influence of economic and policy factors has led to strong regional differences in the development level of Chinese border cities. As a composite dynamic system, the level of urban resilience varies significantly with regional development under various disturbance factors. Therefore, in this study, we use the TOPSIS method to construct a systematic analysis framework for determining the resilience of border cities. Spatial analysis is then used to analyze the spatio-temporal patterns of resilience development for 45 border cities in China from 2010 to 2020. Three representative sample zones are selected to further analyze the spatial differences in border city resilience. The purpose is to explore macro-regional differences in the resilience of Chinese border cities, hoping to provide guidance for the healthy development of China's border cities in the future and provide theoretical reference for government management.

## 2. Concept of border city resilience

Originally conceived in the field of physics as the ability of an object to return to its initial state, the concept of resilience was introduced to ecology by Holling in the 1970s and has since been cited in psychology, engineering, and socioeconomic research (24). After ICLEI-Local Governments for Sustainability formally introduced the concept of resilience to urban issues in 2002, research on urban resilience and the concept of resilient cities have spread worldwide (25). With the increasing complexity of urban development disturbance factors, urban resilience research has gradually become a hot topic in society and academia (26). Currently, the study of urban resilience has become an important way of exploring and addressing sustainable urban development issues in regional economics and geography by closely integrating the “human–land” territorial relationship and the “economic–social–ecological” coupled system. Existing urban resilience research shows multi-disciplinary and multi-disciplinary cross-fertilization, with research methodologies shifting from traditional mathematical and physical characterization to spatial analysis (27). The research area has also become more comprehensive, covering all levels from national to provincial, regional, local municipalities, and villages and towns (28).

**Figure 1:**
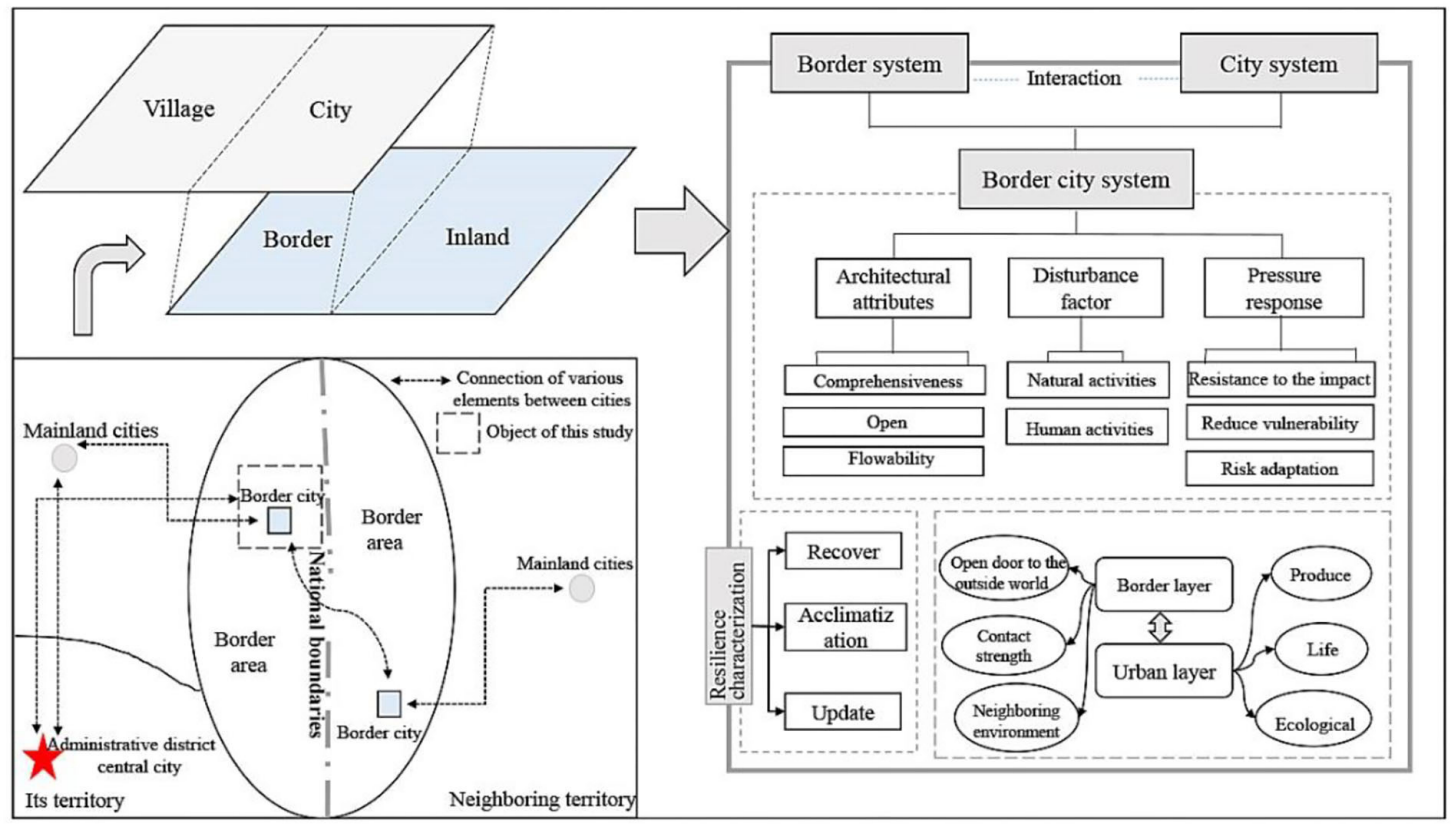
Conceptual representation of border city resilience.

The concept of border city resilience encompasses the state and degree of adaptation of the border city territorial system in overcoming the adverse disturbances caused by the interaction between changes in the natural environment and human activities, based on the advantages of its own structural characteristics and functional attributes. As location conditions carry all the elements necessary for the creation and development of a city, dynamic changes in location conditions inevitably affect the economic, social, and planning development of a city in multiple ways (29). The border is one of the country's most important locational environments, and border cities, as a special type of city, carry the important function of the country's external links, with mutual feedback between the locational conditions of the border and the development of the city. As a result, therefore, the relationship between urban development and location in national border areas is often stronger than that in central cities. Border cities, when viewed as independent systems, also have the systemic property of resilience. Moreover, material transport, information transfer, and energy exchange between the country's inland hinterland and border crossings and between villages and cities in border areas are more frequent and intense than general cities. As an important branch of human–territory relations, the spatial system of border cities must co-ordinate various elements to strengthen development of the urban system and cope with pressures while also linking the internal and external aspects under the border system, absorbing policies, and overcoming disturbances from neighboring countries. In summary, the resilience of border cities can be strengthened or weakened by changes in the ability of the border city system to recover, adapt, and renew itself. At a deeper level, this is reflected in complex interactions between the geographic space of the border, with the border at its core, and the productive space of human settlements, with the city at its core (Figure 1).

## 3. Methodology

### 3.1. Overview of the study area

China has the longest land border (~22,800 km) and the largest number of neighboring countries in the world. The geopolitical development of China's border areas is complex, with widely varying locational conditions. China has 14 land neighbors and 45 prefecture-level border cities in nine border provinces and regions, including Liaoning, Jilin, Heilongjiang, Inner Mongolia, Gansu, Xinjiang, Tibet, Yunnan and Guangxi (Table 1).

**Table 1:** Geographical divisions of border provinces and cities in China.

**Geographical area**	**Border provinces**	**Border city**
Northeast border	Heilongjiang province	Daxing'anling, Heihe, Yichun, Hegang, Jiamusi, Shuangyashan, Jixi, Mudanjiang
	Jilin province	Yanbian Korean Autonomous Prefecture, Baishan, Tonghua
	Liaoning province	Dandong
The northern frontier	Inner Mongolia autonomous region	Hulunbuir, Xing'an League, Xilin Gol League, Ulaan Chab, Baotou, Bayannur, Alashan League
Northwest frontier	Gansu province	Jiuquan
	Xinjiang Uygur autonomous region	Hami, Changji Hui Autonomous Prefecture, Altay Prefecture, Tacheng Prefecture, Bortala Mongolian Autonomous Prefecture, Ili Kazak Autonomous Prefecture, Aksu Prefecture, Kizilsu Kirgiz Autonomous Prefecture, Kashgar Prefecture, and Hotan Prefecture
Tibet border	Tibet autonomous region	Ngari Region, Shigatse, Shannan, Nyingchi
Southwest border	Yunnan province	Nujiang Lisu Autonomous Prefecture, Baoshan, Dehong Dai Jingpo Autonomous Prefecture, Lincang, Pu 'er City, Xishuangbanna Dai Autonomous Prefecture, Honghe Hani and Yi Autonomous Prefecture, Wenshan Zhuang and Miao Autonomous Prefecture
	Guangxi Zhuang autonomous region	Baise, Chongzuo, Fangchenggang

### 3.2. Data sources

Forty-one prefecture-level cities in the border areas of China were selected as the study objects, of which Daxinganling, Tacheng, Ali, and Shannan were excluded from the study owing to serious data deficiencies. The three time points chosen for the study were 2010, the first year of the 12th Five-Year Plan; 2015, the closing year of the 12th Five-Year Plan; and 2020, the end of the 13th Five-Year Plan. The data were mainly obtained for the period 2010–2020 from the China Urban Statistical Yearbook 2010–2020, the Urban Construction Statistical Yearbook of the Ministry of Housing and Construction (http://www.mohurd.gov.cn/xytj/tjzljsxytjgb/jstjnj/), statistical yearbooks of the border provinces and regions, and statistical bulletins of the cities (http://www.tjcn.org/tjgb/) for data supplementation. Data on government-issued policies were obtained from the Ministry of Commerce of the People's Republic of China, the Office of Port Management of the General Administration of Customs, and a list of key border areas in the Opinions on Several Policy Measures to Support the Development and Opening of Key Areas along the Border issued by the State Council. The Country Vulnerability Index was obtained from the *Fund for Peace* website. The UN Human Development Index is based on the United Nations Development Program. Geographical distances were measured using the Baidu map pickup co-ordinate system and ArcGIS10.2 software. Missing data were interpolated to complete the dataset.

### 3.3. Composite evaluation system and model construction

#### 3.3.1. Evaluation indicator system

According to border city-related research and the concept of urban resilience in different fields, we constructed a composite system for evaluating the resilience level of Chinese border cities from two aspects: the city system and the border system. Specifically, we included 21 and 8 indicators at the city and border system levels, respectively, giving a total of 29 indicators (Table 2). The border city resilience index (BCR) was used to characterize the resilience level of each border city in China. A positive or negative BCR indicates whether each indicator increases or decreases the resilience of the border cities. The specific indicators are described below.

**Table 2:** Evaluation index system of border city resilience.

**System**	**Sub-systems**	**Elemental indicators**	**Weighting**	**Properties**
Urban system	Production sub-system	Total GDP/billion RMB (X_1_)	0.0338	+
		GDP per capita/(RMB/person) (X_2_)	0.0323	+
		Year-end urban and rural savings deposit balance/billion RMB (X_3_)	0.0331	+
		Share of tertiary sector in GDP/%(X_4_)	0.0298	+
		Annual tourism revenue/billion RMB (X_5_)	0.0355	+
		Share of science and technology expenditure in fiscal expenditure/% (X_6_)	0.0339	+
		Share of education expenditure in fiscal expenditure/% (X_7_)	0.0389	+
		Volume of goods transported by road/million tons (X_8_)	0.0305	+
	Living sub-system	Total urban resident population/person (X_9_)	0.0380	+
		Number of undergraduates enrolled/person (X_10_)	0.0302	+
		Natural population growth rate/% (X_11_)	0.0293	+
		Unemployment rate/% (X_12_)	0.0328	–
		Social insurance index^①^ (X_13_)	0.0292	+
		Number of beds in medical institutions per 10,000 population/unit (X_14_)	0.0299	+
		Annual road passenger traffic/million passengers (X_15_)	0.0297	+
		Proportion of households with internet broadband access/% (X_16_)	0.0309	+
	Ecological sub-system	Sulfur dioxide emissions per square kilometer/(tons/*KM*^2^) (X_17_)	0.0320	–
		Greenery coverage in built-up areas/% (X_18_)	0.0344	+
		Household waste disposal rate/% (X_19_)	0.0337	+
		Density of drainage pipes in built-up areas/(*km*/*KM*^2^) (X_20_)	0.0316	+
		Integrated utilization rate of industrial solid waste/% (X_21_)	0.0298	+
Border system	Openness to the outside world sub-system	Total imports and exports of border cities/US$ billion (X_22_)	0.0413	+
		Actual amount of foreign investment utilized in border cities/US$ million (X_23_)	0.0828	+
		Number of foreign visitors received by border cities in a year/million (X_24_)	0.0373	+
		Border cities receive strong national policy support^②^ (X_25_) 1. The state council has approved the opening of a class of ports to the outside world. 2. Key development and opening-up pilot zones. 3. National ports along the border. 4. Border Economic Co-operation Zone (BECZ). 5. Cross-border economic cooperation zones.	0.0394	+
	Neighboring countries environment sub-system	Neighboring fragile states index (X_26_)	0.0308	–
		Neighboring countries UN human development index (X_27_)	0.0294	+
	Contact strength sub-system	Distance of border towns from the center of the administrative region/*km* (X_28_)	0.0302	–
		Distance of border cities from border crossing cities of neighboring countries/*km*^③^ (X_29_)	0.0297	–

① The social insurance index indicates the number of city residents with various types of insurance as a proportion of the total urban population.

② For measuring the strength of the city's support by national policies, a value of 1–5 was assigned according to the above five policy conditions, each corresponding to one point.

③ The important border-crossing cities of neighboring countries were selected based on the UN database, foreign trade and economic information from the General Administration of Customs and the statistical yearbooks of each province and region, and the cities with the largest trade links and city size were selected comprehensively.

(1) Urban system layer

According to the urban resilience measurement method, we consider the harmonious symbiosis between production, life, and ecology for three urban spaces. The economic base, resource allocation, and production structure are the basic conditions for the resilience of urban systems. The effectiveness of living space is a fundamental function of a city and a measure of the resilience of the “people” in the city. As a key physical element, the city infrastructure is a direct and real factor influencing the quality of life of its inhabitants. Ecology builds the green barrier of a city, and the creation of a livable ecological city is crucial for preventing flooding and mitigating pollution. Indicators referring to each of these three spatial layers were used to measure the role that each element of the urban system layer in the border city has on its level of resilience.

(2) Border system layer

From its birth to its development, a border city cannot be separated from the geography of the border area, which is connected to the interior and exterior of the city, forming the basis on which cross-border trade is conducted, with the economic prosperity of the city as the carrier. The degree of openness to the outside world in a border economy oriented toward trade at ports has always been a key concern for the state. According to the law of distance decay in geography, the distance between a border city and its neighboring important cities affects the ease of transmitting its resources. In addition, border areas are bordered by neighbors in a complex and volatile environment, where political and social conflicts and the economic level of other countries can become factors of vulnerability that threaten the country according to the openness of its borders. Therefore, we used a three-layer sub-system of openness, strength of ties, and neighboring environment to measure the role that each element of the border system layer in border cities has on the level of resilience.

#### 3.3.2. Comprehensive measure of border city resilience

As each evaluation index has different levels and orders of magnitude, we adopted the z-score standardization method to process the data in a dimensionless way and eliminate the influence of data with different levels of magnitude on the resilience evaluation. The TOPSIS evaluation model, which is based on entropy weights, was used to measure the resilience of Chinese border cities. Specifically, the standardized data were normalized, the weights of each indicator were determined by the entropy weighting method, and the TOPSIS model was applied to calculate the positive and negative ideal solution distances of the target vector for each year by defining a measure in the target space, where the closeness of the evaluation target to the ideal value was calculated for ranking. Finally, we calculated the resilience evaluation value of each border city. The advantage of the entropy-TOPSIS model is that it is operational and the results are reasonable (30). The level of resilience was quantitatively and objectively evaluated for each border city; see the work of (30) for the specific steps.

### 3.4. ArcGIS spatial analysis and resilience grading of border cities

The BCR was calculated using the above evaluation model. Then, ArcGIS10.2 was used to spatially link the BCR values to the study area in vector format and visualize the spatio-temporal geographic information. The natural breakpoint method takes into account the range and number of elements in each group, as close as possible, and can be used to divide subjects into groups with similar attributes. Based on Jenks' natural breakpoint method, the BCR was divided into five categories based on its magnitude, i.e., lowest, lower, medium, higher, and highest resilience (31). The value range and classification standard of BCR are shown in Table 3.

**Table 3:** Classification standard of comprehensive measure of resilience of border cities in China.

	**BCR value range**	**Classification**
Grade 1	< 0.286	Lowest resilience
Grade 2	0.287–0.323	Lower resilience
Grade 3	0.324–0.362	Medium resilience
Grade 4	0.363–0.409	Higher resilience
Grade 5	≥0.410	Highest resilience

### 3.5. Sample zones and geostatistical trendline methods

The sample zone is a collection of continuous linear study sites that represent a series of sites that vary regularly or show significant differences in geographical gradients owing to dominant factor drivers (32). To reveal differences in the spatial patterns of resilience for border cities in typical regions, we selected three sample zones in this study: the northeast region bordering Mongolia, Russia, and North Korea; the Xinjiang region bordering Central Asia; and Guangxi and Yunnan provinces bordering Southeast Asian countries. These sample zones were used to characterize differences in the resilience of border cities in Northeast, Northwest, and Southwest China, respectively. Trendline analysis, which is an analytical method that uses ArcGIS as the analysis platform to plot research data in three-dimensions (X, Y, and Z directions) by projection, is the projection of values into a scatter plot on the X and Z planes and a scatter plot on the Y and Z planes. Think of it as a horizontal view formed from 3D data, and then fit the scatter plot with polynomials on the projection plane, was employed to further reveal the spatial development trend of urban resilience in typical border areas in China.

## 4. Results

### 4.1. Spatio-temporal characteristics of resilience in Chinese border cities

**Figure 2:**
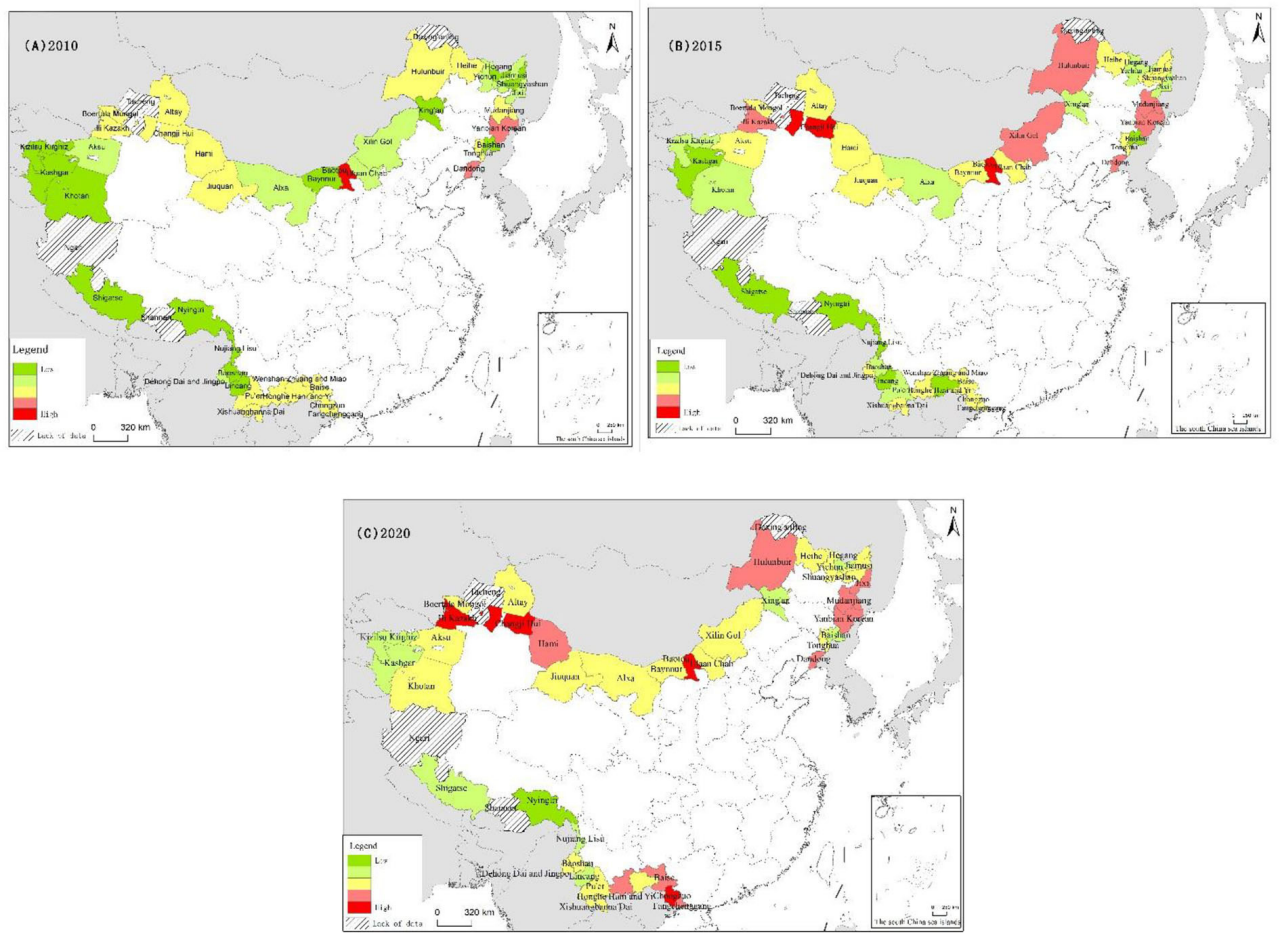
Spatial pattern of resilience for border cities in China in 2010 **(A)**, 2015 **(B)**, and 2020 **(C)**.

The BCR of the 45 prefecture-level cities, states, or regions in China's border areas was measured for the three study years (2010, 2015, and 2020) according to the model described in Section 3.3.2. The average BCR of border cities was 0.3174, 0.3323, and 0.3397 in 2010, 2015, and 2020, respectively, showing a gradual increase in resilience over time, the average resilience of China's border cities in 2015 was 4.694% higher than that in 2010, and in 2020, it was 2.227% higher than that in 2015. When comparing individual BCR values across the 3 years, Xinjiang Changji Prefecture had the highest BCR of 0.5048 in 2015, whereas Kashgar, Xinjiang, had the lowest BCR of 0.2449 in 2010 (Supplementary Table 1). Figure 2 shows the spatial distribution of the five BCR levels for border cities in China.

In 2010, the resilience levels of most border cities in China were generally low, with minimal regional differences. According to the five major regions (Northeast China, Southwest China, Northwest China, North China (Inner Mongolia Autonomous Region and Gansu Province), and Tibetan China), the average resilience level of border cities in 2010 decreased in the following order: North China (0.3222), Northeast China (0.3128), Southwest China (0.3053), Northwest China (0.3043), and Tibetan China (0.2604) (Supplementary Table 2). In terms of their overall typological characteristics, the single “highest-resilience” city was observed in the north (Baotou), whereas “higher-resilience” cities were located in the northeast, namely Yanbian Korean Autonomous Prefecture and Dandong City. “Medium-resilience” cities were concentrated in the northwest region of the northern border and in the southwest region of southeastern Yunnan, extending to Guangxi; all other cities showed a low level of resilience.

The BCR values of border cities in 2015 were significantly different from those in 2010, with all cities showing an overall increase in resilience and more pronounced spatial differences, specifically in the northeast and northwest regions and in eastern Inner Mongolia. That is, cities in northeast and eastern Inner Mongolia showed significant increases in BCR values, whereas the southwest and Tibetan regions showed no significant improvement in resilience. During this year, border cities began to make a breakthrough in terms of resilience quantity and quality, transitioning from only one highest-resilience city and two higher-resilience cities in 2010 to three highest-resilience cities and six higher-resilience cities (Supplementary Table 1, Figure 2).

The resilience level of border cities in 2020 showed a relatively small change from that in 2015, except in the southwest region. The highest-resilience region was dominated by eastern Northeast China, northern Xinjiang, and western Guangxi, whereas the lowest resilience region included southwestern Xinjiang and Tibet, which showed no notable improvement effect. In Yunnan Province, the resilience level of border cities was significantly increased, with a transition from lower resilience to highest resilience and significant regional differences overall. The northern region had the largest average BCR of 0.3514, showing an increase of 0.011 from that in 2015, with the best overall regional resilience quality and the fastest rate of resilience improvement in Tibet, followed by the southwest region, with BCR values showing an increase by 11.4 and 5.52%, respectively, from 2015. In 2020, there were five border cities with the highest resilience and nine border cities with higher resilience. In the northeast, the cities of Jixi, Mudanjiang, Yanbian Korean Autonomous Prefecture, and Dandong had an average resilience index value of 0.3868. The northern region had the most balanced resilience level, with Baotou exhibiting the highest intra-regional resilience level of 0.447, which was decreased from that in 2015. The greatest variation in city resilience levels was found within the northwest region, with the highest resilience level in Yili at 0.4345 and the lowest resilience level in Kizilsu Kirgiz Autonomous Prefecture at 0.299, with a difference of 0.135 between the two, reflecting the huge gap in development between the north and south of Xinjiang. The resilience index of Yunnan border cities in the Tibetan and southwest regions is rising slowly, whereas cities in Guangxi Province have improved significantly, with the emergence of Chongzuo as a high-resilience border city (Supplementary Tables 1, 2).

Regarding the evolution of border city resilience from 2010 to 2020 in more detail (Figure 2), the resilience levels of the four major regions outside Tibet increased overall; however, individual cities within the region showed a decline in resilience. The three regions of Northeast China, the eastern part of Inner Mongolia, and the northern border, which developed significantly faster than other cities in 2010–2015, became the core regions of highest resilience in China. The period during 2015–2020 then marked a major breakthrough in the level of resilience of border cities in the southwest, with border cities in Yunnan Province making a leap from the lowest resilience to medium–high resilience and border cities in Guangxi Province transitioning from medium to high resilience across the board. In this decade, the western part of Inner Mongolia province, the southern part of Xinjiang province, and the Tibet Autonomous Region still showed low levels of resilience, making it difficult to form highest-resilience cities in these regions. In addition, the gap between the resilience levels of border cities gradually widened, and individual cities with high resilience continued to improve while expanding outward, forming a regional trend whereby high-resilience cities formed the nodes that spread to the periphery, whereas the original large clusters of low-resilience cities struggled to further develop their resilience.

### 4.2. Typical sample zones of resilience in Chinese border cities

**Figure 3:**
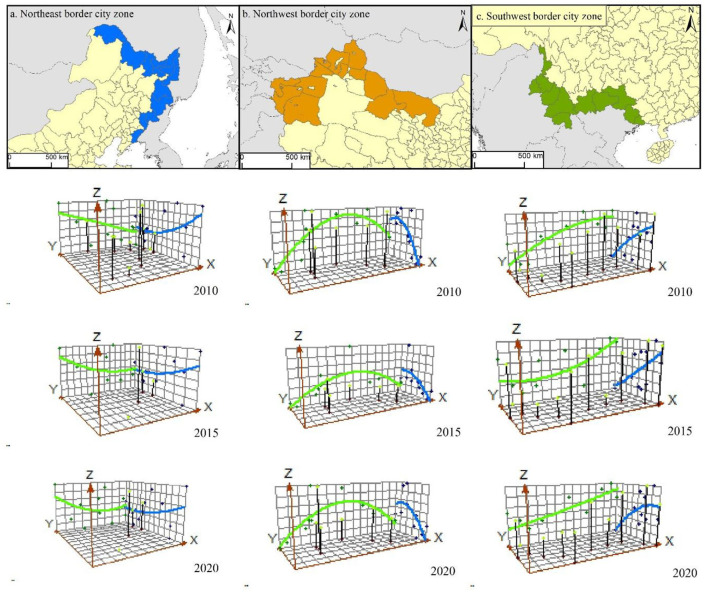
Trend lines showing the resilience level of China's border cities in three sample zones in 2010 **(a)**, 2015 **(b)** and 2020 **(c)**.

In this study, three sample zones of border cities in Northeast China, Xinjiang, and Yunnan–Guangxi, which are located in three key development directions of China's border area, were selected to draw a trend line of the resilience level of each zone in 2010, 2015, and 2020 using the geostatistical trend line analysis method in ArcGIS 10.2 (Figure 3). This analysis further elucidated regional differences in the resilience of border cities in China from the perspective of the spatial gradient of geographical factors.

(1) Northeast China: Northeast Asia sample zone

The BCR values of northeast border cities, which span the three provinces of Heilongjiang, Jilin and Liaoning, were approximately arranged in an inverted “U” shape, i.e., BCR values were higher for the western side, mainly Heihe, the northeast side, including Jixi, Mudanjiang and Yanbian Korean Autonomous Prefecture, and the southern side, Dandong, than for the other cities. However, the three cities on the northeastern side have maintained high levels of resilience over time, increasing their differences from their neighbors and becoming more structurally prominent. Subject to the influence of the urban base and international environment, the northern side of Heilongjiang Province, as the border river between China and Russia, has convenient transportation and serves as a good basis for cooperation between the two countries. Jixi City and Mudanjiang City have their own urban volumes, which are greater than those of the surrounding cities, and have relied in recent years on the advantages of transportation, population, and national policies to improve the quality of development, in addition to port trade, foreign investment, and border tourism to optimize the economic structure, which has accelerated the resilience of these cities. The border cities in the north and east, as traditional forest and agricultural areas in the northeast, have good basic conditions for the development of agriculture and animal husbandry but a weak foundation for secondary and tertiary industries and face the dilemma of industrial structure defects and difficulties in transformation. Moreover, this part of the region has long belonged to the labor export outflow area and faced the pressure of negative population growth, with a high degree of aging and a high unemployment rate, relative to the cities in the northeast and Dandong City in the south, where the export of trade-oriented industrial development is constrained. Intra-regional development disparities have accelerated factor flows, with resources from cities with differing infrastructure flowing more to developed regions, large provincial cities, and the parts of neighboring countries that border China far from their own economic and political centers, which makes it difficult for Northeast China's border cities to prosper. In addition, the long-term population exodus, the existence of social problems such as aging urban infrastructure, low income of residents, and employment difficulties, as well as the low degree of openness of the neighboring Russian Far East and North Korea to the outside world, which reduces the development potential of border cities on the Chinese side, have led to large spatial differences in resilience of the border cities and unstable development trends in this sample zone.

(2) Xinjiang province: Central Asia sample zone

This zone includes border cities with high levels of resilience, consisting of various regions, cities, states, and leagues in northwest China, as well as those bordering Central Asian countries and Mongolia, including Xinjiang, Jiuquan in Gansu, and the Alashan League in Inner Mongolia. The difference in resilience between cities in the east and west of the sample zone was not significant, and its trend line is approximately a smooth curve, characterized by a “convex” structure with low levels in the east and west and high levels in the center. The difference in resilience levels between cities was greatest in 2010 and least prominent in 2015; in 2020, the curve became more pronounced, reflecting an increased difference in resilience levels between cities. This phenomenon is primarily caused by constraints of the natural environment. The northwest region straddles arid and semi-arid zones, with large areas covered by grasslands, deserts, the Gobi, and other land cover types that are relatively harsh. However, Xinjiang, Gansu, and Inner Mongolia rely on their rich resources of livestock, minerals, oil, and gas, coupled with increasing openness toward Central Asian countries, four of which joined the SCO as early as 2001 and have a high degree of political mutual trust. Moreover, closer economic cooperation under the Belt and Road Initiative will, to a certain extent, make better use of the city's proximity to the border and make up for the inherent shortcomings of its natural geographical environment. Furthermore, the ecological environment of the southern border region and western Inner Mongolia still deserves protection, as overgrazing and reclamation of pastureland has led to ecological fragility and consequent severe weather conditions such as sand and dust storms, which directly threaten the normal functioning of the city. In addition, the economic development of the northern border region has long relied on mining mineral resources and petrochemical processing, resulting in pollution and a fragile industrial structure. We recommend that the region as a whole should restore its ecology while developing tourism and that multiple initiatives should be taken to improve the resilience of the city and achieve healthy and sustainable development of northwestern border cities.

(3) Yunnan–Guangxi: Myanmar–Laos–Vietnam sample zone

The sample zone extends from the Nujiang Lisu Autonomous Prefecture in Yunnan to Fangchenggang City in Guangxi and includes 11 cities bordering the Southeast Asian countries of Myanmar, Laos, and Vietnam. The resilience level of border cities in this sample zone increased from west to east, with an overall trend line of double smooth curves in parallel. The region covers three water systems, namely the Nujiang, Lancang, and Duijiang Rivers; cities in the Nujiang River Basin showed the lowest level of resilience, followed by cities in the Lancang River Basin, with cities in the “Youjiang” River Basin in Guangxi Province exhibiting the highest resilience. The Yunnan–Guangxi border area has good water and temperature conditions, high forest cover, and complex topography. Unlike the two sample zones mentioned above, the Yunnan–Guangxi sample zone lacks large areas of flat land conducive to urban-scale development, which limits the carrying space for urban production and living. In this environment, the inconvenience of geographic location becomes a challenging factor that hinders the comprehensive resilience of the city in terms of accessibility and the quality of life of residents. The trend line in 2020 was flatter than that in 2010, with the overall gap narrowing as the resilience of border cities in the southwest region increased across the board. Notably, the strength of national policy support for Yunnan and Guangxi provinces has reinforced the effective functioning of border cities across the border. We found an obvious role of each city in trade with Myanmar, Laos, and Vietnam. Moreover, these cities have attracted increasing attention in terms of good prospects for radiating ASEAN countries, as well as investment in talent, investment environmental management, and other related elements. This has led to an improvement in the quality of urban construction while actively promoting comprehensive construction of border cities with high levels of resilience.

## 5. Discussion

### 5.1. Research contributions and new discoveries

This study makes two major contributions to the research field. First, regarding theoretical knowledge, we construct a comprehensive, focused, and visual system for exploring the resilience and healthy development of border cities in China. This study differs from existing research in relation to the location conditions and development of border cities in China. Most existing studies start from macro-scale countries (regions), meso-scale cities (33), and micro-scale communities (ports, towns, etc.) and involve four main aspects: (1) Local urban problems at the border by focusing on certain categories of elements, such as the environment (34), crime (35), and immigration (36), which are prominent conflicts in the border area. (2) Research on major international and regional issues in border cities is often characterized by the global impact and problem-oriented issues, such as epidemic prevention and control in China–Russia border cities (23). (3) Urban connection in border areas, which focuses on geographical location characteristics, emphasizing the interaction between neighboring cities in the national border, such as the “twin Cities” development model (37). Most of these studies are policy-oriented, and national and local government policies play crucial roles. (4) Micro-scale studies on a specific border city, including ports and small border towns. This type of research can examine almost all the developmental elements of border cities and provide substantial development guidance and suggestions for local governments with local orientation (22).

In this study, we summarize the methods and processes of these four categories of research, which represent studies of “cities in the border” (38) and “the border outside the city” (39), to easily split the connection between border areas and urban spaces. Our research embodies the dual structure of “urban–rural” and the spatial mode of “border–inland,” based on research into the border–city relationship (40), and starts from the concept of resilience, considering the three aspects of structure attributes, disturbance factors, and pressure response in the border city system and then constructs a comprehensive evaluation system of China's border cities. This model not only quantitatively evaluates the resilience of border cities in various periods and regions in China but also identifies weak items through data weights and visually determines the advantages and disadvantages of border cities from an overall perspective, which can be used to propose relevant regional development policies. According to the empirical measurements of this system, we showed that the resilience level of border cities differs significantly among different regions of China and has obvious temporal fluctuations. According to the characteristics of sample zones, different inter-regional border cities have different structures. In the future, this model can also be used to make development predictions and explore the functional combinations of border cities.

The second major contribution of this study is to provide guiding suggestions for the development of Chinese border cities in combination with government policies. The national government provides policy support to border cities, and local governments take measures to formulate effective development models according to local conditions. In this study, we explored the weaknesses of regional border cities through resilience measures and spatial differences in recent years and proposed measures to improve urban resilience. These measures include maintaining high-resilience border cities to optimize the urbanization structure, reasonably optimizing the urbanization quality, building a border area growth pole, actively undertaking the industrial transfer of developed areas, introducing emerging industries, paying attention to technological innovation, improving the product market and competitiveness, creating a good market investment environment, developing a series of investment promotion preferential policies to enhance city vitality, encouraging scientific and technological innovation, and promoting the development of border city transformation measures (41). For border cities with low resilience and stagnant upward trends, smart development strategies are required to improve the quality of development, for example, controlling the scale of urban land use, ecological reconstruction, strengthening social security, improving urban service functions, improving the happiness of urban residents, and relying on a good ecological environment and rich historical, cultural, and natural landscape to develop tourism. During the development of border city tourism, the industry should strengthen the construction of tourism service infrastructure, actively develop diversified tourism products, integrate tourism resources, strengthen publicity around their tourism resources, and improve the visibility and attraction of tourism resources. Furthermore, appropriate measures should be taken to promote the high-quality development of urban resilience. For various regional border cities in China, comprehensively improving the resilience of cities is an important guarantee for achieving sustainable urban development, improving infrastructure construction, and coordinating regional development. Moreover, to achieve complementary advantages between cities, measures are required to improve the resilience of border cities, such as attracting talent and appropriately encouraging fertility (42). In particular, the port economy in border cities maintains the status and growth of resilience, with ports making a vast economic contribution to the economic development of border cities; therefore, port development measures should explore new border trade preferential policies and consider policy changes with neighboring countries to ensure positive countermeasures.

### 5.2. Study limitations

The limitations of this study are predominantly related to data collection limitations and the complex factors affecting the resilience of border cities. As border cities are far less comprehensive than mainland capital cities and coastal cities in terms of data disclosure, the lack of data is a serious issue. Moreover, the reality of China's border cities is complex. China's border areas are vast, and border cities in different regions are faced with different levels of economic development and resource endowments; therefore, it is difficult to identify a unified development pattern. Although we deconstructed the concept and analytical framework of the resilience of China's border cities and then analyzed their spatio-temporal patterns and regional differences, we did not further explore the factors influencing the resilience of border cities because of objective factors such as the complexity of the main body of border cities and the difficulty of obtaining some data. We aim to conduct further research on this aspect in future. The development of China's border areas has attracted substantial attention from the international community and Chinese government. Various international, national, and local incentive policies have been introduced to encourage development (20, 43). In future, the development of this special region should be more closely related to the geopolitical environment and should build on the conclusions of this study through additional field research, dialogue, and interviews.

## 6. Conclusion

In this study, we collected urban panel data for 2010, 2015, and 2020, as well as data used to characterize internal and external city linkages, to analyze the spatio-temporal characteristics and evolution of resilience in border-level cities in China at the national scale. A city resilience system was constructed from the perspective of border city relationships, and the main interannual variations and spatial differences in the resilience of China's border cities were summarized by quantifying their resilience levels. The main findings are as follows:

1. We observed significant spatial divergence in the level of resilience in Chinese border cities, with overall resilience decreasing the following order: Northeast China > Southwest China > Northwest China > North China > Tibetan China. Coastal border cities such as Dandong and Fangchenggang had greater resilience than inland border cities. The spatial differences in BCR values were consistent with the economic level of border regions as well as the construction of ports and export-oriented trade orientation. Moreover, the spatial difference coincides with the level of economic and trade development of border areas, which directly influences investment in urban infrastructure and environmental management. The economy is not only an important component of the border system but also the basis for increased resilience of the urban system at border locations. However, gaps in the resilience level of border cities are inextricably linked to factors such as physical geography, historical foundations, population movements, and policies. Most border cities in China still rely on their geographical location and geopolitical advantages. In addition to forming an economic development model that focuses on foreign trade, close links between the main body of the city and the border crossing and its surrounding areas are strengthened. By ensuring the healthy and high-quality development of highly resilient cities, the quality of resilience in small- and medium-sized cities can be improved, and the geographical gap in resilience between cities can be gradually reduced.
2. From 2010 to 2020, the overall resilience level of China's border cities showed an upward trend, with spatial disparities gradually increasing. In particular, 2010–2015 was a critical period for the resilience of border cities nationwide, and by 2015, the combined resilience of northeast China and the northern border region of northwest China had reached its highest level, becoming the most resilient areas of China. In 2020, the number of cities with medium and high levels of resilience dominated; however, concentrated contiguous areas of cities with medium to low levels of resilience still existed. For border cities, the elements between the border and urban systems cannot be separated, and the integration and matching of elements between systems is key to improving the resilience of border cities.
3. According to its geographical proximity, the northeastern sample zone showed an inverted “U” shape, with western, northeastern, and southern parts exhibiting high and continuously increasing resilience and all other cities in the zone exhibiting reduced resilience. The Xinjiang sample zone showed no significant differences from east to west, and the resilience trend line was approximately a smooth curve, characterized by a “convex” structure with low levels in the east and west and high levels in the center and a “gentle to prominent” trend over time. The Yunnan–Guangxi sample zone showed an increasing level of resilience from west to east, with a double smooth curve parallel structure. Cities in different sample zones and stages of development face significantly different risks and shocks. The construction of a resilient city at the border must be tailored to the local conditions, that is, the regional nature of the border and the stage of urban development. Simultaneously, refined and dynamic coping strategies that consider the general environment and future urban development potential of border areas should be developed.

## Data availability statement

The original contributions presented in the study are included in the article/Supplementary material, further inquiries can be directed to the corresponding author.

## Author contributions

LS: form analysis, topic research, method proposal, progress management, and article writing. FP: fund acquisition, conception, supervision, review, and editing and modification suggestions. SW: conceptualization, methodology, theory proposal and verification, software application and visualization analysis, article writing, and data management. All authors contributed to the article and approved the submitted version.
