# Supplementary material for: Spatio-temporal evolution of the resilience of Chinese border cities
Source: Front Public Health. 2022 Dec 20;10:1101799. doi: 10.3389/fpubh.2022.1101799 (PMC9807667; doi:10.3389/fpubh.2022.1101799)
Supplement: Supplementary file 1 [file Table_1.DOCX]

Supplementary Material

# Supplementary Tables

1.1 **Supplementary Table 1.** The resilience of China's border cities in 2010,2015 and 2020.

| **City** | **2010** | **2015** | **2020** | **City** | **2010** | **2015** | **2020** |
| --- | --- | --- | --- | --- | --- | --- | --- |
| Heihe | 0.319728 | 0.346456 | 0.362231 | Ili Kazak | 0.341781 | 0.389514 | 0.434469 |
| Yichun | 0.289272 | 0.304813 | 0.339853 | Aksu | 0.291062 | 0.336212 | 0.354677 |
| Hegang | 0.27422 | 0.301062 | 0.313426 | Kizilsu Kirgiz | 0.254135 | 0.293508 | 0.299013 |
| Jiamusi | 0.299765 | 0.330257 | 0.348517 | Keshgar | 0.244972 | 0.271536 | 0.312363 |
| Shuangyashan | 0.259884 | 0.328101 | 0.329661 | Khotan | 0.260867 | 0.314507 | 0.336121 |
| Jixi | 0.297337 | 0.312405 | 0.368694 | Shigatse | 0.261737 | 0.273897 | 0.316728 |
| Mudanjiang | 0.340411 | 0.398479 | 0.396822 | Nyingtri | 0.256534 | 0.261493 | 0.283083 |
| Yanbian Korean | 0.354663 | 0.368734 | 0.373004 | Nujiang Lisu | 0.261723 | 0.269214 | 0.285591 |
| Baishan | 0.269864 | 0.275059 | 0.311444 | Baoshan | 0.270207 | 0.297627 | 0.342395 |
| Tonghua | 0.318574 | 0.347707 | 0.335254 | Dehong Dai and Jingpo | 0.299502 | 0.335522 | 0.361392 |
| Dandong | 0.353546 | 0.39451 | 0.408563 | Lincang | 0.267734 | 0.285087 | 0.322967 |
| Xing'an | 0.265171 | 0.288822 | 0.311288 | Pu’er | 0.306442 | 0.293608 | 0.337025 |
| Hulunbuir | 0.311397 | 0.365926 | 0.372777 | Xishuangbanna Dai | 0.301049 | 0.329416 | 0.344695 |
| Xilin Gol | 0.295444 | 0.367579 | 0.341481 | Honghe Hani and Ni | 0.317589 | 0.341773 | 0.389901 |
| Ulaan Chab | 0.291268 | 0.331106 | 0.353916 | Wenshan Zhuang and Miao | 0.338481 | 0.282712 | 0.34665 |
| Baotou | 0.378016 | 0.43461 | 0.446975 | Baise | 0.318777 | 0.340381 | 0.404188 |
| Baynnur | 0.270968 | 0.33796 | 0.359723 | Chongzuo | 0.311227 | 0.359149 | 0.419747 |
| Alxa | 0.281696 | 0.311077 | 0.327601 | Fangchenggang | 0.338829 | 0.353012 | 0.38075 |
| Jiuquan | 0.338088 | 0.343173 | 0.355138 |  |  |  |  |
| Hami | 0.315082 | 0.332958 | 0.378799 |  |  |  |  |
| Changji Hui | 0.332218 | 0.504755 | 0.426371 |  |  |  |  |
| Altay | 0.30945 | 0.334044 | 0.348363 |  |  |  |  |
| Boertala Mongol | 0.311597 | 0.34544 | 0.353724 |  |  |  |  |

1.2 **Supplementary Table 2.** Average resilience of border cities in different zones of China.

| **Zone** | **2010** | **2015** | **2020** | **Improvement in 10 years** |
| --- | --- | --- | --- | --- |
| Northeast | 0.3128 | 0.3318 | 0.3470 | 0.0342 |
| North | 0.3222 | 0.3403 | 0.3514 | 0.0293 |
| Northwest | 0.3043 | 0.3377 | 0.3441 | 0.0397 |
| Tibet | 0.2604 | 0.2676 | 0.3020 | 0.0417 |
| Southwest | 0.3053 | 0.3183 | 0.3369 | 0.0315 |
